# Tau acetylation at K331 has limited impact on tau pathology *in vivo*

**DOI:** 10.1002/1873-3468.70320

**Published:** 2026-03-10

**Authors:** Shoko Hashimoto, Yukio Matsuba, Mika Takahashi, Takaomi C. Saido

**Affiliations:** ^1^ Pioneering Research Division, Medical Innovation Research Center Shiga University of Medical Science Otsu Shiga Japan; ^2^ Laboratory for Proteolytic Neuroscience, RIKEN Center for Brain Science Wako Saitama Japan

**Keywords:** acetylation, Alzheimer's disease, knock‐in mouse models, post‐translational modifications, Tau

## Abstract

Post‐translational modifications regulate tau aggregation and propagation, yet how amyloid pathology shapes the tau modification landscape remains unclear. Using liquid chromatography–tandem mass spectrometry, we compared tau modifications in *MAPT* knock‐in (*MAPT* KI) mice and *MAPT*/*App* double knock‐in mice with *App* ^*NL‐G‐F*^ amyloid pathology. Only subtle differences were detected, with a tendency toward increased acetylation within the repeat domain. Because K321 and K331 lie in the fibril‐forming core, their roles were further examined. Acetylation at these sites was absent in cynomolgus monkey brains. To test functional relevance, we generated acetylation‐mimicking *MAPT* ^K331Q^ knock‐in mice on the *MAPT* KI background. Despite tau expression, these mice showed reduced tau phosphorylation at 24 months, with unchanged insoluble tau and seeding activity. Thus, K331 acetylation does not promote tau pathology.

## Graphical abstract

We mapped tau post‐translational modifications in humanized *MAPT* knock‐in mice and in amyloid‐bearing double knock‐in mice. Acetylation within the repeat domain, particularly around K331, showed modest increases under amyloid pathology. To test functional relevance, we generated *MAPT* ^K331Q^ knock‐in mice. K331 acetylation did not enhance tau phosphorylation, aggregation, or seeding, indicating no pathogenic impact *in vivo*.

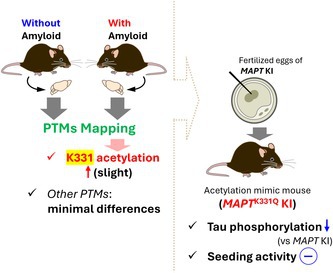

## Abbreviations

**Aβ**, Amyloid‐β

**APP**, Amyloid precursor protein

**FRET**, Förster resonance energy transfer

**IP**, Immunoprecipitation

**LC–MS/MS**, Liquid chromatography–tandem mass spectrometry

***MAPT* KI**, Human *MAPT* knock‐in mouse.

***MAPT*** ^**K331Q**^ **KI**, Acetylation‐mimicking *MAPT* K331Q knock‐in mouse.

**PTM**, Post‐translational modification

**RD**, Repeat domain (of tau)

**sgRNA**, Single‐guide RNA

**ssODN**, Single‐stranded oligodeoxynucleotide

Alzheimer's disease is the most common type of dementia. The pathological features of Alzheimer's disease in postmortem brains include amyloid plaques formed by aggregated amyloid‐β and neurofibrillary tangles that are composed of hyperphosphorylated tau [1, 2]. Such abnormal protein aggregation leads to neuronal dysfunction and cognitive decline. Tau aggregation can be observed occasionally in the brains of elderly individuals with normal cognitive function (referred to as primary age‐related tauopathy). However, in such cases, tau pathology remains localized to restricted regions, and it is not accompanied by evident neurodegeneration [3]. By contrast, if amyloid pathology is present, tau pathology spreads rapidly throughout the brain. Indeed, in Alzheimer's disease progression, amyloid pathology initially emerges, followed by widespread tau pathology, ultimately leading to neurodegeneration and cognitive impairment. Experimental studies have further shown that the introduction of tau aggregation seeds into model mice with amyloid pathology accelerates the propagation of tau pathology [4, 5]. However, the mechanism by which amyloid pathology promotes the formation of tau pathology remains unclear.

Amyloid pathology promotes tau phosphorylation [5, 6, 7, 8]. In addition to phosphorylation, tau undergoes other post‐translational modifications (PTMs), and changes in these modifications under amyloid pathology are likely to influence tau aggregation and propagation. Indeed, several studies have performed comprehensive analyses of tau PTMs. Wesseling et al. [9] performed high‐resolution profiling of tau PTMs in the brains of patients with Alzheimer's disease and healthy controls. Results showed that tau PTMs in Alzheimer's disease brains exhibited significant heterogeneity across patients and disease stages. Nevertheless, some modifications such as specific phosphorylation and acetylation events occur at high frequency. Similarly, Zola et al. [10] reported that several disease‐specific PTM combinations, including ubiquitination, acetylation, and phosphorylation, were identified in soluble tau, and these combinations were strongly correlated with the isoform composition of tau aggregates. These findings indicate that tau PTMs are important for disease progression and may contribute to disease specificity. Nonetheless, no single pathogenic PTM has been identified yet. Morris et al. [11] mapped tau PTMs in wild‐type and hAPP transgenic mice. They found that both models had similar PTM profiles, emphasizing the difficulty of identifying disease‐driving modifications. Notably, this study analyzed mouse endogenous tau. Taken together, these findings underscore the importance of examining PTM changes in models expressing human tau under amyloid pathology, where the system provides a clearer context to detect disease‐relevant modifications.

The current study established a PTM mapping method using *MAPT* knock‐in (*MAPT* KI) mice [12, 13], which express human tau under physiological regulation and recapitulate the coding sequence and isoform composition of humans. To investigate how amyloid pathology alters tau modifications, PTM mapping was performed on double KI mice generated by crossing *MAPT* KI mice with *App* ^*NL‐G‐F*^ KI mice, which developed amyloid pathology [8]. Further, to assess the conservation of these modifications in species more closely related to humans, brain samples from cynomolgus monkeys were also analyzed. Results showed that acetylation at lysine 331 within the tau repeat domain (RD) was a modification possibly influenced by amyloid pathology. To directly evaluate its physiological significance, acetylation‐mimicking KI (*MAPT* ^K331Q^ KI) mice were generated, and the effects of this modification on tau phosphorylation and aggregation *in vivo* were analyzed.

## Materials and methods

### Animals

The Wako Animal Experiment Committee of RIKEN (approval number: W2021‐2‐020(1)) and the Management Committee of the Research Center for Animal Life Science at Shiga University of Medical Science (approval number: 2023‐7‐5) approved the animal experiments. All experiments were conducted in compliance with the following guidelines: Regulations for the animal experiments (RIKEN), Wako Institute Animal Experiment Handbook (RIKEN), Guidelines for the Husbandry and Management of Laboratory Animals of Research Center for Animal Life Science at Shiga University of Medical Science, and the ARRIVE guidelines 2.0.

Mice were maintained under controlled temperature (22 ± 2 °C) and humidity (50 ± 10%) with a 12 h light/dark cycle and had free access to food and water. Detailed information on the animals used in each experiment, including sex and number, is provided in Table S1. The *App* ^*NL‐G‐F/NL‐G‐F*^ KI (*App* ^*NL‐G‐F*^) mice were previously generated using genomic DNA corresponding to introns 15–17 of the mouse *App* gene, which humanized the amyloid‐β sequence and introduced the KM670/671NL (Swedish), I716F (Iberian), and E693G (Arctic) mutations [8]. In addition, we established the human *MAPT* KI (*MAPT* KI) mice, in which the coding region of the *MAPT* sequence was replaced with the human *MAPT* sequence [12, 13]. Mutant *MAPT* KI mice (*MAPT* ^+3^ KI, *MAPT* ^S305N+3^ KI, and *MAPT* ^P301S+3^ KI) harboring FTDP‐17 mutations achieved via genome editing based on *MAPT* KI mice were recently developed [14, 15].

### Development of *MAPT* ^K331Q^ KI mice

The K331Q mutation was introduced into the humanized *MAPT* KI background, in which the endogenous mouse *Mapt* coding sequence is replaced by the human *MAPT* coding sequence. *MAPT* ^K331Q^ KI mice were generated using the CRISPR/Cas9 gene‐editing technology. The template DNA required for RNA synthesis was established using oligo DNA that contains the target sequence, along with a forward primer containing a T7 promoter sequence and a CRISPR tail reverse primer. After generating this template, sgRNA was transcribed with the MEGAshortscript T7 Transcription Kit (Thermo Fisher Scientific, San Jose, CA, USA) and subsequently purified using the MEGAClear Transcription Clean‐Up Kit (Thermo Fisher Scientific). Cas9 mRNA was transcribed and polyadenylated using the mMESSAGE mMACHINE T7 ULTRA Transcription Kit (Thermo Fisher Scientific) using the pX330 vector as a template. The single‐stranded oligodeoxynucleotide (ssODN) carrying the K331Q mutation was used for homologous recombination. Cas9 mRNA, sgRNA, and ssODN were microinjected into the *MAPT* KI mouse zygotes. These zygotes were incubated at 37 °C in 5% CO_2_ until they reached the two‐cell stage, during which they were transferred to the recipient mice. The Support Unit for Animal Resource Development at RIKEN CBS performed microinjection and mouse breeding procedures. Genomic DNA was extracted from tail and brain tissues using a standard ethanol precipitation method. DNA fragments for sequencing were amplified by polymerase chain reaction using the following primers: forward, 5′‐ACATTTTTCCACCCTGACTAGGA‐3′; reverse, 5′‐TATTTCACTTCACTCCCGCCTC‐3′. Genomic DNA was amplified using the KOD FX Neo DNA polymerase (Toyobo, Osaka, Japan). Sanger sequencing was performed using the forward primer and polymerase chain reaction product with the BigDye Terminator v3.1 Cycle Sequencing Kit (Thermo Fisher Scientific).

All experiments described in this study were performed using homozygous *MAPT* KI–based mouse lines, including *MAPT* ^K331Q^ KI mice, to minimize variability due to gene dosage effects.

### Immunoprecipitation and sample preparation for mass spectrometry

The Tau13 antibody (sc‐21 796, Santa Cruz Biotechnology, Dallas, TX, USA) was covalently conjugated to Dynabeads M270 Epoxy (Thermo Fisher Scientific), according to the manufacturer's instructions, using 5 μg of antibody with 1 mg of beads. For immunoprecipitation, protein samples were prepared from the cortical tissues of *MAPT* KI, *App* ^*NL‐G‐F*^ × *MAPT* KI, or wild‐type cynomolgus monkeys. Frozen tissues were homogenized in 50 mm Tris–HCl (pH 7.5) containing a protease inhibitor cocktail and a phosphatase inhibitor cocktail. After centrifugation at 20 000 ***g*** for 20 min at 4 °C, the resulting supernatants were collected, and protein concentrations were measured. Next, 2.5 mg (250 μL) of antibody‐coated beads were transferred to a new tube, and the supernatant was removed using a magnetic stand. The beads were washed once with 200 μL of homogenization buffer. Next, a protein solution at a concentration of 1 mg·mL^−1^ was added to the beads. The mixture was incubated on a roller at 4 °C for 1 h. After incubation, the beads were washed three times with 200 μL of Tris‐buffered saline (TBS). After the supernatant was removed, the beads were incubated on a roller with TBS containing 0.02% Tween‐20 to remove any remaining unbound proteins. The beads were then washed three times with 200 μL of TBS to eliminate Tween‐20. To elute the proteins, 60 μL of 0.1% trifluoroacetic acid was added to the beads, suspending them in solution. After incubation for 5 min on a roller at room temperature, the supernatant was collected. To neutralize the supernatant, 18 μL of 50 mm triethylamine was added. From this sample, 10 μL was taken for western blot analysis and silver staining. The remaining sample was dried using a centrifugal evaporator, and 100 μL of 50 mm ammonium bicarbonate and 11 μL of CH_3_CN (with a final CH_3_CN of 10%) was added. Subsequently, 2 μL of 1 m dithiothreitol solution was added (with a final dithiothreitol concentration of 20 mm), and the mixture was incubated at 56 °C for 30 min. After returning to room temperature, 10 μL of 333 mm iodoacetamide solution was added (with a final iodoacetamide concentration of 30 mm), and the mixture was incubated in the dark at 37 °C for 30 min. Finally, trypsin was added at an enzyme‐to‐substrate ratio of 1:50, and the mixture was incubated overnight at 37 °C. The resulting product was then used for mass spectrometry (MS) analysis.

### Mass spectrometry

MS was performed at the Research Resource Division, RIKEN Center for Brain Science. The prepared protein fragments were analyzed using a liquid chromatography system (EASY‐nLC 1000; Thermo Fisher Scientific, Odense, Denmark) coupled with the Q Exactive hybrid quadrupole‐orbitrap mass spectrometer (Thermo Fisher Scientific) equipped with a nanospray ion source operating in positive mode. The peptides derived from the protein fragments were separated on the NANO‐HPLC C18 capillary column (0.075 mm inner diameter × 150 mm length, particle size of 3 μm; Nikkyo Technos, Tokyo, Japan). The mobile phase ‘A’ comprised water containing 0.1% formic acid, and the mobile phase ‘B’ involved acetonitrile with 0.1% formic acid. A 60‐min gradient was applied with two different slopes at a flow rate of 300 nL·min^−1^, from 5% to 35% B over 48 min, followed by 35% to 65% B over 12 min. The Q Exactive MS was operated in the top‐10 data‐dependent acquisition mode. The instrument parameters were as follows: spray voltage, 2.3 kV; capillary temperature, 275 °C; mass range, 350–1800 m/z; and normalized collision energy, 28%. Raw data were acquired using the Xcalibur software.

### Protein identification

MS and MS/MS data were analyzed using the MASCOT software (Matrix Science, London, the UK) to search against the Swiss‐Prot database. The search parameters for MASCOT were defined as follows: Trypsin was set as the enzyme, and carbamidomethylation of cysteines was specified as a fixed modification. Then, methionine oxidation was allowed as a variable modification. The peptide mass tolerance was set to ±1.5 Da, with a fragment mass tolerance of ±0.8 Da. A maximum of one missed cleavage was permitted. For PTM mapping, variable modifications, including lysine acetylation, lysine ubiquitination, and serine/threonine/tyrosine phosphorylation were additionally included in the search parameters. The identified PTM sites were validated based on ion scores, manual inspection of MS/MS spectra, and reproducibility across biological replicates. A comprehensive list of all tau peptides identified by LC–MS/MS, including both modified and unmodified peptides, is provided in Table S2. A corresponding comprehensive list of tau peptides identified from the cynomolgus monkey sample is provided in Table S3.

### Silver staining

The gel after sodium dodecyl sulfate‐polyacrylamide gel electrophoresis was stained according to the attached protocol using Sil‐Best Stain One (Nacalai Tesque, Kyoto, Japan). Briefly, after fixation with the fixative solution (45% methanol and 5% acetic acid), the gel was treated with 10% ethanol and then reacted with the silver staining solution. After rinsing, the bands were developed using the developing solution, and the reaction was stopped with 5% acetic acid.

### Western blot analysis

An equivalent amount of protein from each animal was mixed with a 4x sample buffer. The protein samples were separated via sodium dodecyl sulfate‐polyacrylamide gel electrophoresis and transferred to a polyvinylidene difluoride membrane (Merck Millipore, Burlington, MA, USA) using electrophoretic transfer. The membrane was treated with enhanced chemiluminescence prime blocking solutions (Cytiva, Marlborough, MA, USA) and reacted with each antibody (Table S4) diluted in blocking buffer overnight at 4 °C. Thereafter, the membrane was washed three times with TBS with Tween‐20 for 10 min and then incubated with horseradish peroxidase‐conjugated anti‐rabbit or anti‐mouse IgG (Cytiva) for 1 h. The immunoreactive bands on the membrane were visualized using ECL Select (Cytiva) and scanned with the LuminoGraph I (Atto, Tokyo, Japan).

For quantification, the major full‐length human tau bands detected by AT8 and PHF1 antibodies were quantified. Band intensities were normalized to β‐actin as a loading control, and the same band positions were used consistently across all samples. Multiple tau bands detected by AT8, PHF1, and Tau5 reflect different phosphorylation states and isoforms of human tau expressed from the *MAPT* knock‐in allele.

### Immunohistochemistry

Fixed paraffin‐embedded brain sections (4 μm) were deparaffinized and subjected to antigen retrieval by autoclaving at 121 °C for 5 min in 10 mm sodium citrate buffer (pH 6.0). Endogenous peroxidase was quenched with 0.3% hydrogen peroxide in methanol, and sections were blocked with 0.2% casein in PBS. Primary antibodies (listed in Table S4) diluted in PBS were applied overnight at 4 °C. After washing, sections were incubated with Alexa Fluor 488‐ or 555‐conjugated secondary antibodies (1:500, Molecular Probes, Eugene, OR, USA). Stained sections were coverslipped with ProLong Gold Antifade Mountant (Thermo Fisher Scientific) and imaged using a BZ‐X series microscope (Keyence, Osaka, Japan) at 10× magnification.

### Tau FRET biosensor assay (HEK293T RD‐CFP/YFP cells)

Human embryonic kidney 293T (HEK293T) Förster resonance energy transfer (FRET) biosensor cells stably expressing tau RD‐CFP/YFP were used for tau seeding assays. This cell line corresponds to Tau RD P301S FRET Biosensor (ATCC CRL‐3275; RRID: CVCL_DA04) and was maintained in Dulbecco's Modified Eagle Medium (high glucose) with 10% fetal bovine serum and 100 unit·mL^−1^penicillin/100 μg·mL^−1^streptomycin at 37 °C in a humidified atmosphere of 5% CO_2_. The Tau RD P301S FRET Biosensor line was derived from HEK293T cells by transduction with lentiviral constructs encoding tau RD P301S‐CFP and tau RD P301S‐YFP and cloned following dual positive cell sorting [16]. All cell lines used in this study were authenticated within the past 3 years using short tandem repeat (STR) profiling. STR profiles were compared with reference databases to confirm cell line identity. Cells were routinely tested for mycoplasma contamination using a PCR‐based detection assay, and all experiments were performed using mycoplasma‐free cells. For the assays, the cells were seeded at 35 000 cells per well in 96‐well plates (500 μL·well^−1^) and allowed to adhere overnight. Tris–HCl‐soluble brain fractions (cortex) collected from the *MAPT* KI, *MAPT* ^K331Q^ KI, and PS19 mice (positive control) were quantified by bicinchoninic acid. For each well, 10 μg of total protein was mixed with Lipofectamine 3,000, according to the manufacturer's protocol, and added. Control wells received Tris–HCl buffer plus reagent only. After incubation for 24 h at 37 °C, the cells were detached with 0.05% trypsin‐ethylenediaminetetraacetic acid, fixed in 2% paraformaldehyde for 10 min at room temperature, washed with phosphate buffered saline, and passed through a 40‐μm strainer to remove clumps. Flow cytometry was performed on BD LSRFortessa X‐20 (BD, Franklin Lakes, NJ, USA). Cells were first gated by forward scatter (FSC)‐A/side scatter (SSC)‐A to exclude debris and then by FSC‐H/FSC‐W to select singlets. Viable and fluorescently positive populations were identified using FITC (YFP) and BV421‐A (CFP) signals. Finally, FRET‐positive cells were defined on BV510‐A (FRET) versus BV421‐A (CFP) bivariate plots, using PS19‐derived material as a positive control and single‐color or vehicle samples for compensation. For each sample, 20 000 singlet events were collected. The percentage of FRET‐positive cells was calculated per well and summarized as mean ± standard error of the mean (*n* = 3 biological replicates).

### Statistical analyses

All analyses were performed using GraphPad Prism 10 (San Diego, CA, USA). The Student's *t*‐test was used to assess the statistical significance of between‐group differences.

## Results

### Identification of post‐translational modification in tau using *MAPT* KI mice

**Fig. 1:**
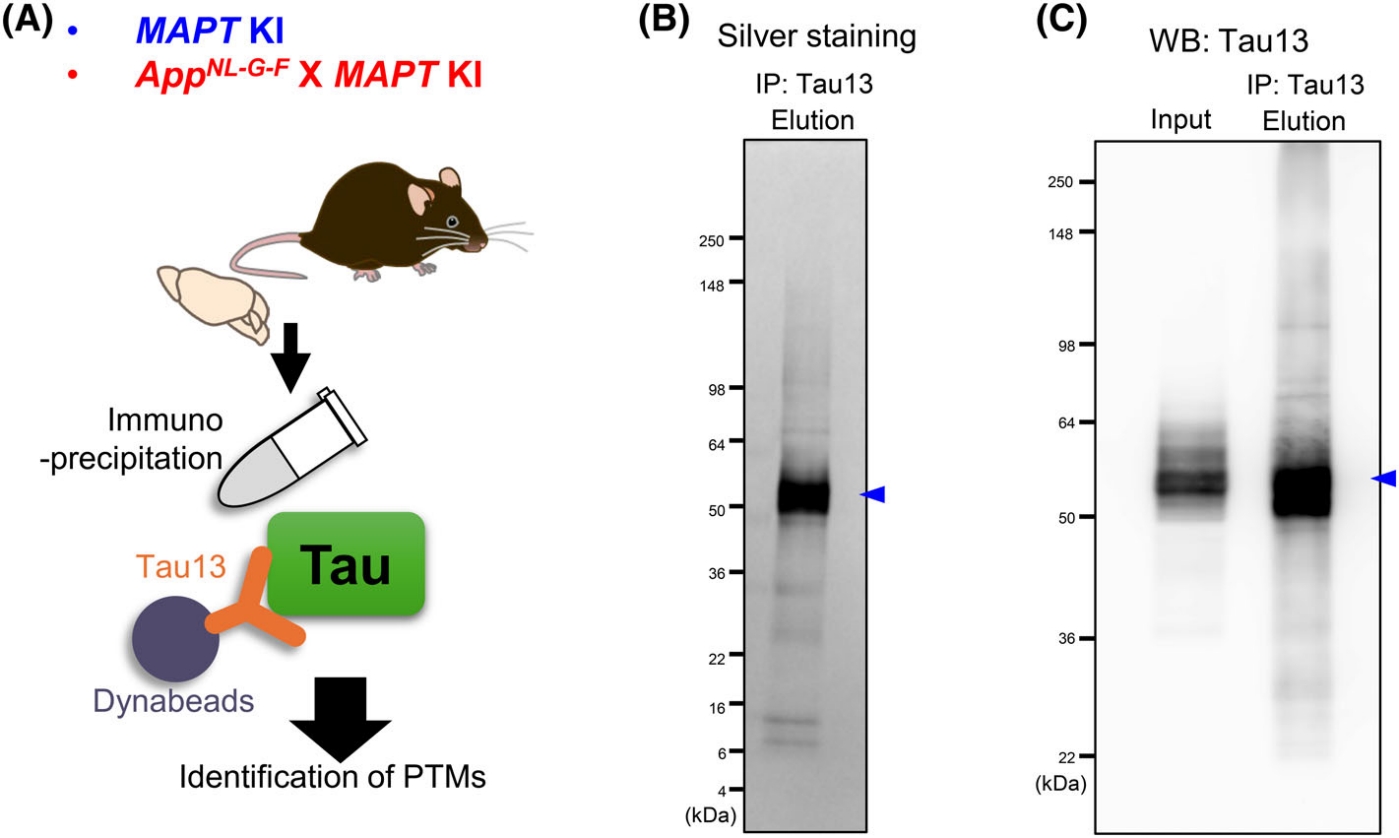
Establishment of post‐translational modifications (PTM) mapping of tau. (A) Schematic overview of the PTM mapping workflow. Tau was immunoprecipitated using the Tau13 antibody (anti‐human tau antibody) from the Tris–HCl buffer soluble fraction of cortical and hippocampal tissues derived from 20‐ to 24‐month‐old female *MAPT* KI and *App* ^*NL‐G‐F*^ X *MAPT* KI mice (*n* = 4 each). PTMs were identified via liquid chromatography–tandem mass spectrometry analysis. (B) Silver staining of immunoprecipitated samples. Tau was observed as the major band. (C) Western blot analysis of input and immunoprecipitated samples using Tau13 antibody, confirming an efficient enrichment of tau.

By identifying and comparing the PTMs of tau under both physiological and pathological conditions, PTMs that significantly contributed to the tau pathology could be identified. The wild‐type mouse could be a simple model for physiological conditions. However, differences in tau sequences and isoform compositions in mice may lead to variations in binding proteins compared with humans [17]. This study established a PTM mapping method using brain samples collected from *MAPT* KI mice, which expressed human‐like tau at a steady level and, similar to humans, had six isoforms of tau expressed in their brains [12]. This allowed for a higher likelihood of identifying physiological modifications. In addition, double KI mice crossed with *App* ^*NL‐G‐F*^ KI mice, which exhibited amyloid pathology, were used to identify PTMs under amyloid pathology [8, 13]. Immunoprecipitation of tau was successfully performed using the Tau13 antibody, which recognizes human tau (Fig. 1A–C, Fig. S1A, 1B). By covalently linking the Tau13 antibody to magnetic beads, the carryover of antibody‐derived fragments into the eluted sample from the beads decreased (Fig. 1B, Fig. S1A).

**Fig. 2:**
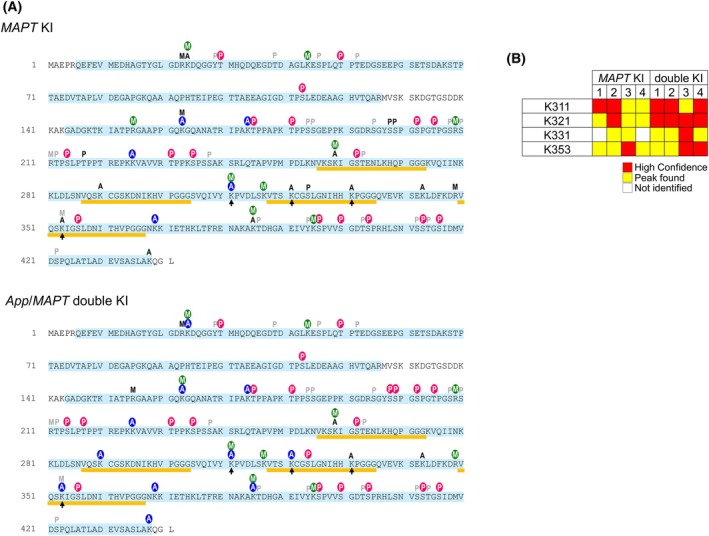
Identification of post‐translational modifications (PTM) of tau via liquid chromatography–tandem mass spectrometry (LC–MS/MS). (A) PTMs identified across the full‐length tau sequence were summarized at the site level based on reproducibility across four biological replicates. Regions highlighted in blue indicate peptides detected by LC–MS/MS. Sites detected in at least two out of four samples with high or medium confidence in at least one peptide are shown as colored filled circles with white letters indicating the PTM type (P, phosphorylation; A, acetylation; M, methylation). Sites detected in at least two out of four samples only with low confidence or as ‘peak found’ (without high or medium confidence in any peptide) are shown as black letter annotations. PTM sites with localization probabilities <75% are indicated in gray font. Data are shown separately for *MAPT* KI and *App* ^*NL‐G‐F*^ × *MAPT* double KI mice. The yellow bar indicates the repeat domain (RD), and the positions of K311, K321, K331, and K353 are indicated by arrows. (B) Acetylation states of lysine residues within the RD in each individual mouse. Numbers 1–4 represent individual animals. The red marks denote high‐confidence acetylation, and the yellow marks represent low‐confidence acetylation identified in each sample.

Figure 2A summarizes the PTM sites identified across the full‐length tau sequence in *MAPT* KI and *App* ^*NL‐G‐F*^ × *MAPT* double KI mice (*n* = 4 per genotype). Identified sites are displayed separately for each genotype. Sites detected in at least two out of four samples with high or medium confidence are shown as colored filled circles with white letters, whereas sites detected in at least two out of four samples only with low confidence or classified as ‘peak found’ (without high or medium confidence in any peptide) are shown as black letter annotations. Sites with localization probabilities <75% are indicated in gray. Figure S2 shows the detailed list of peptides containing all identified PTMs and the confidence level of detection for each individual sample. Table S2 depicts the complete list of all tau‐derived peptides identified in this study, including their sequences, modification types, and relative abundances. Multiple types of tau PTMs, including phosphorylation, acetylation, methylation, nitrosylation, and ubiquitination, were comprehensively analyzed. However, nitrosylation and ubiquitination were not detected with sufficient confidence at the modification‐site level and were therefore not included in subsequent analyses. When assessing PTMs across *MAPT* KI and double KI mice, all major modifications were present in both lines. These results indicate that even under nonpathological conditions, such as in *MAPT* KI mice, tau undergoes diverse post‐translational modifications. Since this analysis was limited to soluble tau, the possibility that amyloid‐associated PTM changes are more prominent in insoluble fractions could not be excluded. Among the phosphorylation sites identified, residues around Ser396, Ser400, and Ser404, which correspond to the paired helical filament 1 (PHF1) epitope, had a higher abundance in double KI mice compared with *MAPT* KI mice (Table S2). Figure 2B presents the detection status of peptides containing acetylated lysines within the RD, categorized as high confidence, peak found, or not detected. Compared with *MAPT* KI mice, acetylation in this region was detected more consistently and with higher confidence in the double KI mice, indicating that RD acetylation is enhanced under amyloid pathology. In particular, K321 and K331 were more likely to be detected with higher confidence in the double KI mice. These acetylation events have also been reported in fibrils derived from Alzheimer's disease brain tissues [18].

### Comparison of PTMs between *MAPT* KI mice and cynomolgus monkeys

**Fig. 3:**
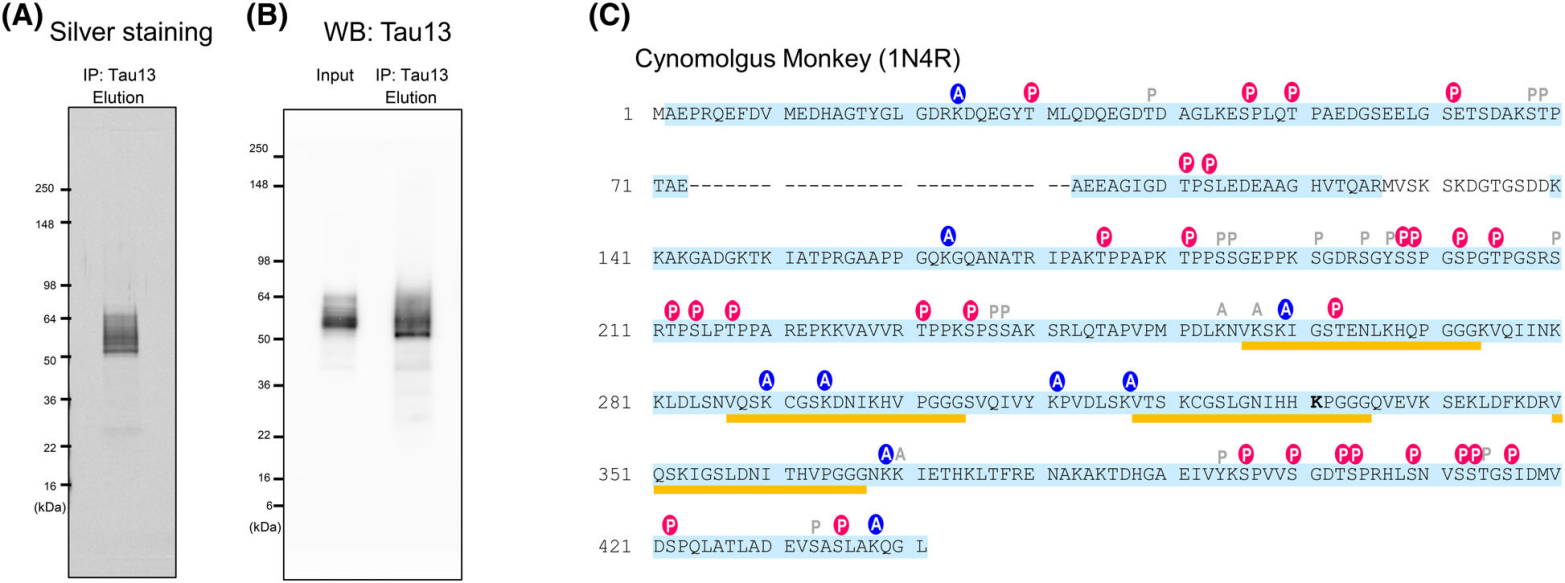
Immunoprecipitation and post‐translational modification (PTM) analysis of tau using cynomolgus monkey brain tissues. (A) Silver staining of immunoprecipitants obtained from cerebral cortex tissue of a wild‐type cynomolgus monkey (*n* = 1) using the same method as in mice. Tau was observed as the major band. (B) Western blot analysis of input and immunoprecipitated samples using the Tau13 antibody, which confirmed enrichment of tau. (C) PTMs identified from the cynomolgus monkey sample (*n* = 1). Regions highlighted in blue indicate peptides detected by liquid chromatography–tandem mass spectrometry (LC–MS/MS). Only phosphorylation and acetylation were analyzed, and peptides covering most of the 1N4R tau isoform sequence were detected. Although the biological sample number was one, LC–MS/MS measurements were performed in technical duplicates, and the integrated results are presented. PTM sites identified with high or medium confidence are represented by colored filled circles with white letters indicating the PTM type (P, phosphorylation; A, acetylation). PTM sites with localization probabilities <75% are indicated in gray font. The regions corresponding to the four repeat domains (RDs) are indicated by yellow lines, and the residue corresponding to human K331 is highlighted in bold.

To validate whether PTMs identified using *MAPT* KI mice are similarly identified in more human‐like animals, a PTM mapping of tau was conducted using brain samples collected from the nonhuman primate, cynomolgus monkey. The cynomolgus monkeys, which belong to the *Macaca genus*, have an amino acid sequence of tau that is extremely similar to that of humans. Tau immunoprecipitation was also successfully performed using the Tau13 antibody on brain samples derived from a cynomolgus monkey (Fig. 3A,B). Phosphorylation sites were significantly conserved between the *MAPT* KI mice and a cynomolgus monkey, with several overlapping modifications observed in both species (Fig. 3C). These observations suggest that overall tau PTM profiles are broadly conserved across species when human tau is expressed, while specific acetylation events such as those at K321 and K331 may depend on pathological or cellular context. In contrast, acetylation at K321 and K331, which showed differential modification between the *MAPT* KI and the double KI mice, was not detected in the cynomolgus monkey brain. This finding could be attributed to the use of wild‐type animals without amyloid pathology.

### Development of *MAPT* KI mice harboring acetylation‐mimicking mutations in the repeat domain

**Fig. 4:**
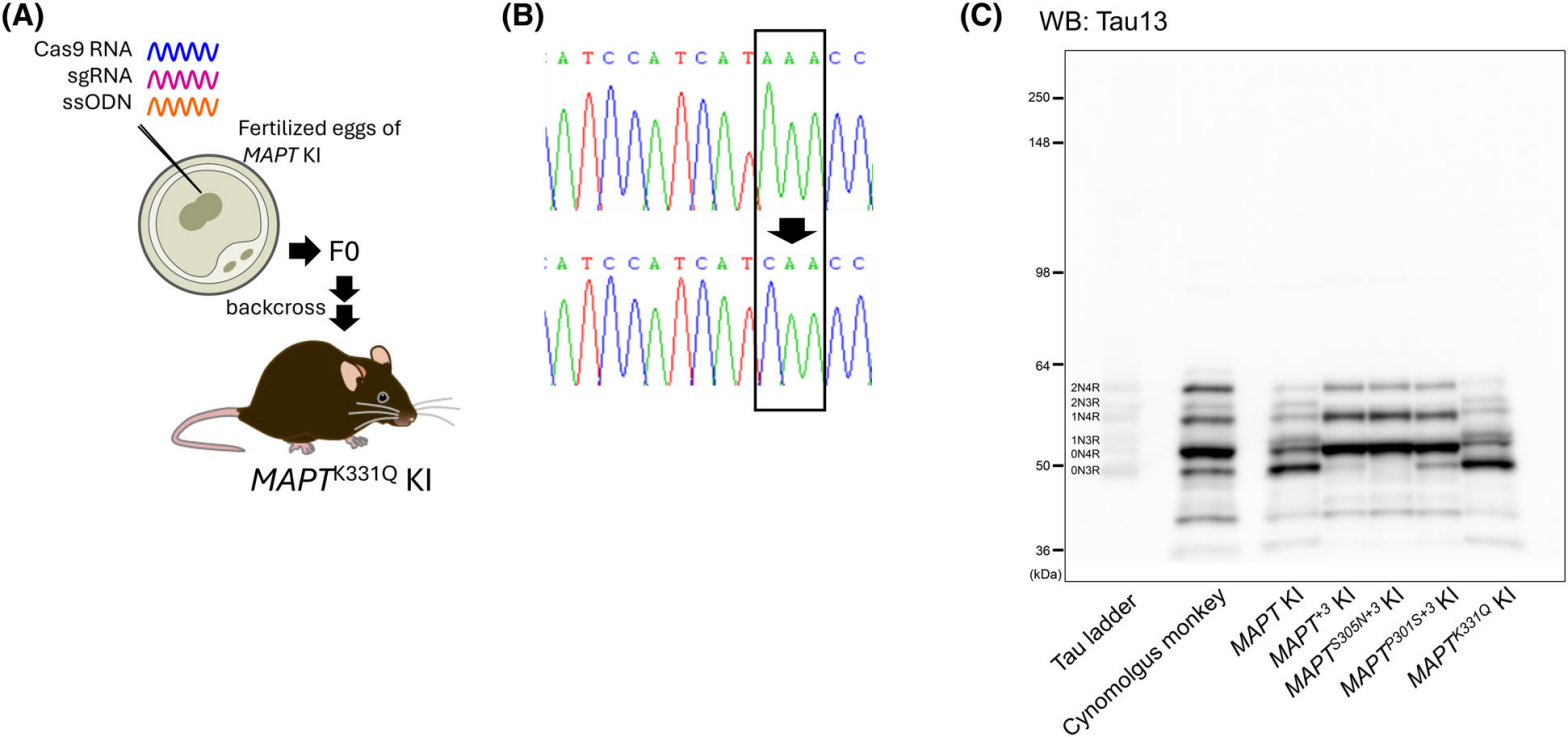
Generation and validation of *MAPT* ^K331Q^ knock‐in (KI) mice and isoform analysis across species and models. (A) Schematic representation of the development of *MAPT* ^K331Q^ mice. Cas9 nuclease mRNA, single‐guide RNA, and single‐stranded oligodeoxynucleotide were injected into the fertilized eggs of *MAPT* KI mice to introduce the K331Q mutation. (B) Sequence analysis of *MAPT* ^K331Q^ KI mice confirmed the substitution of AAA (Lys) to CAA (Gln) at codon 331. (C) Cortical samples collected from cynomolgus monkeys and *MAPT* KI, *MAPT* ^+3^ KI, *MAPT* ^S305N+3^ KI, *MAPT* ^P301S+3^ KI (18 months of age), and *MAPT* ^K331Q^ KI (12 months of age) mice were dephosphorylated and analyzed via western blot analysis using the Tau13 antibody. The banding pattern confirmed the isoform expression ratios of tau in each model.

Next, whether the acetylation of lysine residues within the RD contributes to tau pathology formation was investigated. In particular, we focused on lysine residues K321 and K331, both of which are located within the fibril‐forming core region. K321 lies within a KXGS motif of the RD, a key region for microtubule binding and aggregation [18, 19]. Acetylation‐mimicking substitution at K321 (K321Q) inhibits thioflavin S binding [20]. In contrast, K331 is located near the R3–R4 boundary and close to the aggregation‐prone core motif [18, 19]. Moreover, in Alzheimer's disease‐derived PHFs, K331 contributes to fibril stabilization by forming hydrogen bonds with the opposite protofilament [18, 19, 21]. To examine how the acetylation of these lysine residues influences tau pathology, we attempted to generate acetylation‐mimicking KI mice based on the *MAPT* KI background using CRISPR‐Cas9 genome editing and homologous recombination (Fig. 4A). Figure S3A,B depicts the sequences of the ssODN and sgRNA used and the offspring obtained (including the numbers of indel vs. KI alleles), respectively. Compared with the transgenic or adeno‐associated virus‐mediated overexpression systems, in which expression levels vary and can confound the interpretation of site‐specific mutations, the KI approach provides a physiologically regulated context that enables a more accurate evaluation of the effects of mimic mutations.

As a result, although we attempted to generate a K321 mutant line, this was confirmed to be technically difficult and could not be achieved. Nevertheless, we successfully developed an acetylation‐mimicking KI line for K331 (*MAPT* ^K331Q^ KI) (Fig. 4B). We confirmed that there were no significant differences in the total tau levels or isoform composition between the *MAPT* KI (Wild‐type) and *MAPT* ^K331Q^ KI mice. By contrast, the +3 (harboring the IVS10 + 3 G>A splice‐site mutation), P301S + 3 (harboring the P301S mutation and the IVS10 + 3 G>A splice‐site mutation), and S305N + 3 (harboring the S305N mutation and the IVS10 + 3 G>A mutation) KI lines exhibited a predominance of 4R tau isoforms due to the combined effects of the S305N mutation and the IVS10 + 3 G>A mutation (Fig. 4C). In the cynomolgus monkey brain, four‐repeat tau was predominant. However, three‐repeat tau was also expressed (Fig. 4C).

### *In vivo* effects of K331 acetylation mimic on tau pathology

**Fig. 5:**
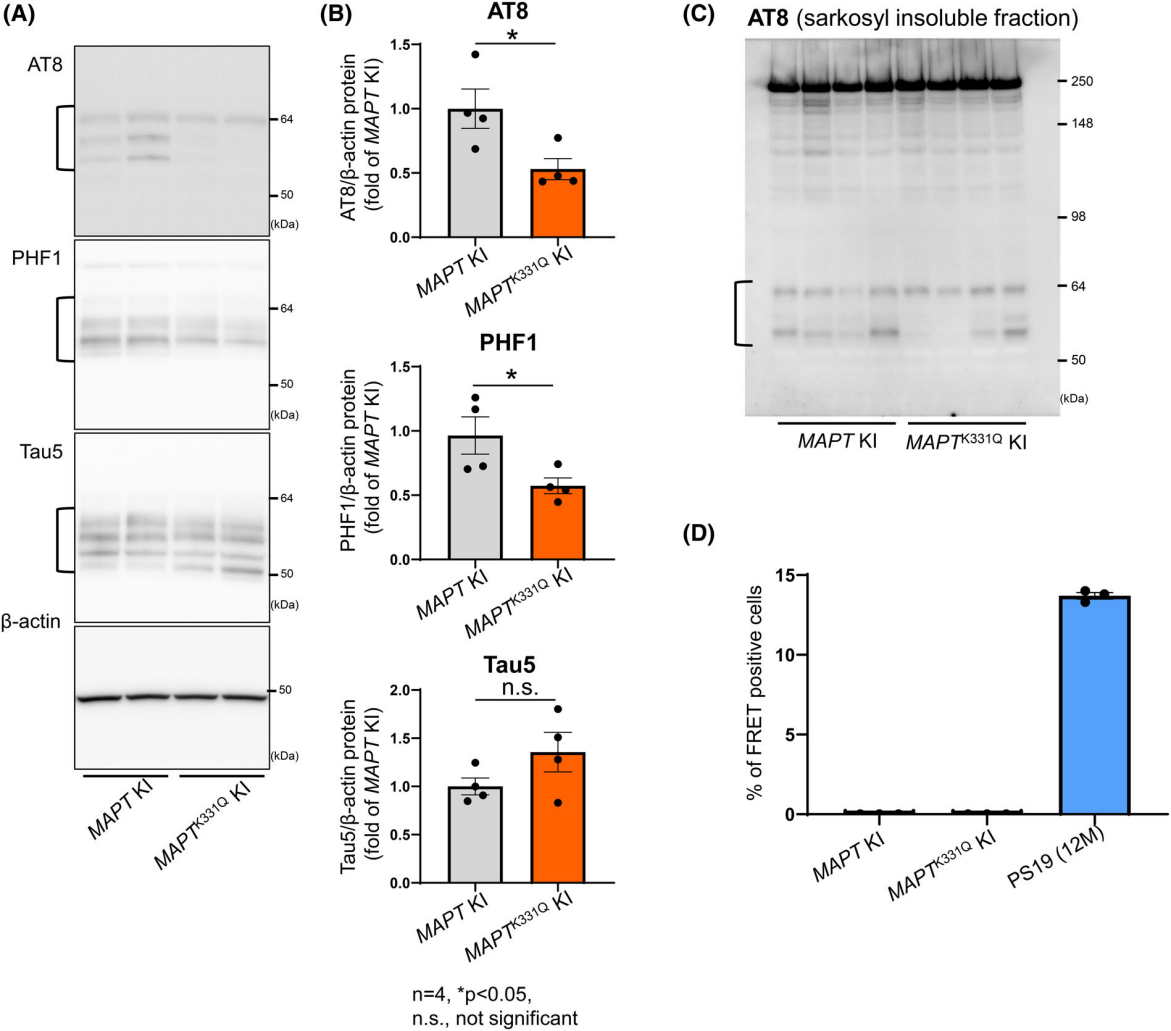
Biochemical and functional characterization of tau in *MAPT* knock‐in (KI) and *MAPT* ^K331Q^ KI mice. Western blot analysis of Tris–HCl soluble fractions from the cortices of 24‐month‐old *MAPT* KI and *MAPT* ^K331Q^ KI mice using AT8 (pSer202/pThr205), PHF1 (pSer396/pSer404), and Tau5 (total tau) antibodies (*n* = 4 per genotype; 2 males and 2 females). (B) Quantification of western blot signals shown in (A). Bands used for quantification correspond to the tau‐positive signals within the bracketed region indicated in the blot. Data are presented as the mean ± standard error of the mean. *P < 0.05. (C) Western blot analysis of 1% sarkosyl‐insoluble fractions prepared from the cortices of 18‐month‐old *MAPT* KI and *MAPT* ^K331Q^ KI mice using the AT8 antibody (*n* = 4 per genotype; all males). Insoluble tau was detected as bands migrating between approximately 50–64 kDa, indicated by a single bracket. Strong bands observed at higher molecular weights were considered nonspecific and were not analyzed. (D) Tau seeding activity measured using the HEK293T Förster resonance energy transfer (FRET) biosensor assay (Holmes et al. [16]). Tris–HCl–soluble fractions from the cortices of 24‐month‐old *MAPT* KI and *MAPT* ^K331Q^ KI (*n* = 3 per genotype; 2 males and 1 female) mice were added to the biosensor cells. Brain lysate from 12‐month‐old PS19 mice (*n* = 3; all males) was included as a positive control to validate assay sensitivity. After 24 h, FRET signals were analyzed by flow cytometry, and the percentage of FRET‐positive cells is shown as bar graphs (*n* = 3 per group). Seeding activity in *MAPT* KI and *MAPT* ^K331Q^ KI lysates was below the detection limit. Data are presented as the mean ± standard error of the mean.

After allowing the *MAPT* ^K331Q^ KI mice to age up to 24 months, the tau phosphorylation state and seeding activity were biochemically analyzed. Consistent with the findings at 18 months (Fig. S4A,B), the *MAPT* ^K331Q^ KI mice exhibited reduced levels of phosphorylated tau compared with the *MAPT* KI mice, as detected on AT8 and PHF1 immunoblotting (Fig. 5A,B). In contrast, the amount of sarkosyl‐insoluble tau did not differ significantly between the two genotypes at 18 months (Fig. 5C). Consistent with these biochemical findings, immunohistochemical analyses revealed no significant differences in microglial or astrocytic reactivity between *MAPT* ^K331Q^ KI and *MAPT* KI mice, as assessed by Iba1 and GFAP staining (Fig. S5). To determine whether acetylation‐mimicking K331Q affects tau seeding activity, we performed a tau seeding assay using CFP/YFP tau fragment‐expressing HEK293T cells. In this assay, cell lysates were added to HEK293T cells stably expressing CFP‐ and YFP‐tagged tau RD fragments, and tau aggregation was detected as FRET‐positive signals by flow cytometry. The percentage of FRET‐positive cells was used as an index of seeding activity. Using this assay, proteins derived from *MAPT* ^K331Q^ KI mice did not promote tau aggregation (Fig. 5D, Fig. S6). PS19 lysate was used as a positive control to confirm assay performance, whereas seeding activity in *MAPT* KI and *MAPT* ^K331Q^ KI lysates was below the detection limit.

Based on our results, at least in the context of the *MAPT* KI background, acetylation at K331 not only failed to exacerbate tau pathology but might have even exerted a suppressive effect on tau phosphorylation. K331 acetylation might have also exerted a protective effect under certain conditions, thereby preventing excessive tau phosphorylation. However, its pathological relevance may emerge only in the presence of additional modifications and mutations or with distinct isoform balance and a longer disease course in the human context. Collectively, these results indicate that acetylation at K331 by itself does not promote tau pathology *in vivo* and may even act as a protective modification. This finding highlights the complexity of site‐specific tau acetylation and the importance of combinatorial or species‐specific factors in determining their pathological impact.

## Discussion

This study established a comprehensive method for mapping tau PTMs using *MAPT* KI mice. Based on a comparative analysis of *MAPT* KI mice and double KI mice that also developed amyloid pathology, no major differences were found in the overall PTM profile. This finding is consistent with the findings of Morris et al. [11], who reported similar PTM signatures in wild‐type and hAPP transgenic mice. However, within the RD lysine residues, particularly K321 and K331, acetylation was more likely to be identified with higher confidence in the double KI mice. To validate the physiological significance of K331 acetylation, *MAPT* ^K331Q^ KI mice were generated, and the effects of this modification in the context of physiologically expressed human tau was directly evaluated. Results showed that the K331Q mice did not show increased tau aggregation or seeding activity. Rather, they exhibited reduced tau phosphorylation at the AT8 and PHF1 epitopes. Taken together, these findings suggest that acetylation at K331 alone may exert a protective effect, rather than promote tau pathology. Further, they highlight the significance of directly assessing site‐specific modifications *in vivo* under the context of physiological expression.

The observed reduction in tau phosphorylation in *MAPT* ^K331Q^ KI mice involved AT8 (S202/T205) and PHF1 (S396/S404), which are epitopes that are typically enhanced during disease progression. This finding suggests that K331 acetylation may attenuate phosphorylation events linked to pathological acceleration. Acetylation had similar protective effects at other residues. For example, acetylation at K321 within the KXGS motif suppresses phosphorylation at adjacent serines [22], and acetylation at K259, K290, and K353 reduces tau aggregation [20]. Taken together, these findings support the idea that certain acetylation events can counteract phosphorylation‐ and aggregation‐driven tau pathology. In contrast, acetylation at K280 is associated with pathological outcomes. The overexpression of p300, which increases K280 acetylation, enhances phosphorylation at S202/T205, T231, and S422. Meanwhile, substitution of K280 with alanine reverses these effects [23]. Moreover, K280 acetylation is associated with increased tau fibrillization and is frequently observed in neurofibrillary tangles in Alzheimer's disease [24, 25]. Recent structural studies have further shown that acetylation at K280 stabilizes fibril formation by creating novel hydrogen‐bonding and hydrophobic interactions within the amyloid motif, thereby directly promoting aggregation [21]. Thus, RD acetylation is not uniformly deleterious. Its impact is highly site‐specific, with some residues such as K321 and K353 counteracting phosphorylation and others such as K280 exacerbate tau pathology. A recent study also revealed that acetylation can act in an isoform‐specific manner, thereby promoting aggregation of three‐repeat tau while inhibiting that of four‐repeat tau [25]. These findings underscore the multifaceted nature of tau acetylation, indicating that its functional consequences may depend not only on the modified residue but also on the isoform context. Therefore, comparison with 4R‐dominant tau KI models (+3, P301S + 3, and S305N + 3) highlights that K331 acetylation alone is insufficient to drive tau pathogenicity under conditions permissive for aggregation.

Although acetylation at K331 has been identified in Alzheimer's disease brains [26], its pathological relevance is likely dependent on broader molecular and pathological contexts. Acetylation at K331 was not detected in cynomolgus monkey brains, which is consistent with the absence of amyloid pathology in these wild‐type animals. This finding indicates that K331 acetylation may arise preferentially under disease conditions. Furthermore, the lack of pathogenic effects in our *MAPT* ^K331Q^ KI mice implies that additional PTMs or genetic factors may be required for acetylation at this site to exert deleterious consequences. For example, RD acetylation along with other modifications or *MAPT* mutations (such as P301S) may act synergistically to destabilize tau. Collectively, these results emphasize that the functional effects of tau PTMs are highly context dependent, influenced by pathological conditions, disease stage, and interactions with other modifications.

The current study had several limitations that should be considered. First, our PTM mapping analysis was limited to the soluble fraction of tau, and insoluble or aggregated species were not comprehensively assessed. As this study represents early and potentially reversible stages of tau modification prior to insoluble aggregation, it focused on soluble fraction. Soluble tau is also more amenable to reproducible mass spectrometric detection, thereby allowing a clearer comparison of PTM profiles under different pathological conditions. Second, *MAPT* KI mice closely recapitulate human tau expression and isoform composition. However, their shorter lifespan and simpler brain organization may limit the long‐term accumulation of PTM effects. Third, our analysis focused on a single acetylation site. Thus, the interplay among multiple PTMs was not addressed. In addition, although comprehensive profiling of tau PTM by IP/MS would provide further mechanistic insight into potential secondary effects of the K331Q modification, such analyses were beyond the scope of the present study. Instead, we prioritized functionally relevant *in vivo* readouts, including tau phosphorylation, insolubility, and seeding competence. Notably, none of these pathological features were enhanced in *MAPT* ^K331Q^ KI mice, suggesting that any potential changes in other PTMs do not translate into increased tau pathogenicity under physiological expression conditions. Hence, future studies that introduce acetylation‐mimicking mutations, such as K331Q and K321Q, into tauopathy models carrying pathogenic *MAPT* mutations, such as *MAPT* ^p301s+3^ KI and *MAPT* ^S305N+3^ KI, may reveal synergistic effects on tau phosphorylation and pathology formation. Finally, our findings have therapeutic implications. In particular, although the global inhibition of tau acetylation has been proposed, our data indicate that certain acetylation events, such as K331 acetylation, may be neutral or even have protective effects. These insights underscore the importance of developing strategies that selectively target pathogenic modifications, rather than presuming that tau acetylation uniformly produces deleterious effects.

Uncropped western blot images are shown in Figs S7–S10.

## Conflict of interest

There are no conflicts of interest to disclose.

## Author contributions

SH designed the research and wrote the manuscript. SH, YM, and MT conducted the experiments. TCS developed the mouse models and provided suggestions for the study.

## Supporting information


**Fig. S1** Full view of the gel corresponding to Fig. 1A.
**Fig. S2.** Summary of all tau peptides and post‐translational modifications (PTMs) identified by LC–MS/MS.
**Fig. S3.** Generation of *MAPT* ^K331Q^ knock‐in mice.
**Fig. S4.** Phosphorylation state of tau in soluble fractions of 18‐month‐old *MAPT* KI and *MAPT* ^K331Q^ KI mice.
**Fig. S5.** Immunohistochemical analysis of microglial and astrocytic reactivity at 24 months of age.
**Fig. S6.** Flow cytometry analysis of tau seeding activity.
**Fig. S7.** Full western blot images corresponding to Fig. 3A and B.
**Fig. S8.** Full western blot images corresponding to Fig. 4C.
**Fig. S9.** Full western blot images corresponding to Fig. 5A.
**Fig. S10.** Full western blot images corresponding to Fig. 5C.

**Table S1.** Summary of animals used in each experiment.

**Table S2.** Comprehensive list of all tau peptides identified by LC–MS/MS, including modified and unmodified peptides.

**Table S3.** Comprehensive list of all tau peptides identified from the cynomolgus.

**Table S4.** Antibodies used for the immunohistochemistry (IHC) and western blotting.

## Data Availability

All data supporting the findings of this study are included in the manuscript and its Supplementary Information.
