# Supplementary material for: Tau acetylation at K331 has limited impact on tau pathology in vivo
Source: FEBS Lett. 2026 Mar 10;600(14):2050–62. doi: 10.1002/1873-3468.70320 (PMC13404147; doi:10.1002/1873-3468.70320)
Supplement: Supplementary file 1 — Fig. S1 Full view of the gel corresponding to Fig. 1A. Fig. S2. Summary of all tau peptides and post‐translational modifications (PTMs) identified by LC–MS/MS. Fig. S3. Generation of MAPT K331Q knock‐in mice. Fig. S4. Phosphorylation state of tau in soluble fractions of 18‐month‐old MAPT KI and MAPT K331Q KI mice. Fig. S5. Immunohistochemical analysis of microglial and astrocytic reactivity at 24 months of age. Fig. S6. Flow cytometry analysis of tau seeding activity. Fig. S7. Full western blot images corresponding to Fig. 3A and B. Fig. S8. Full western blot images corresponding to Fig. 4C. Fig. S9. Full western blot images corresponding to Fig. 5A. Fig. S10. Full western blot images corresponding to Fig. 5C. [file FEB2-600-2050-s004.pdf]

Figure S1

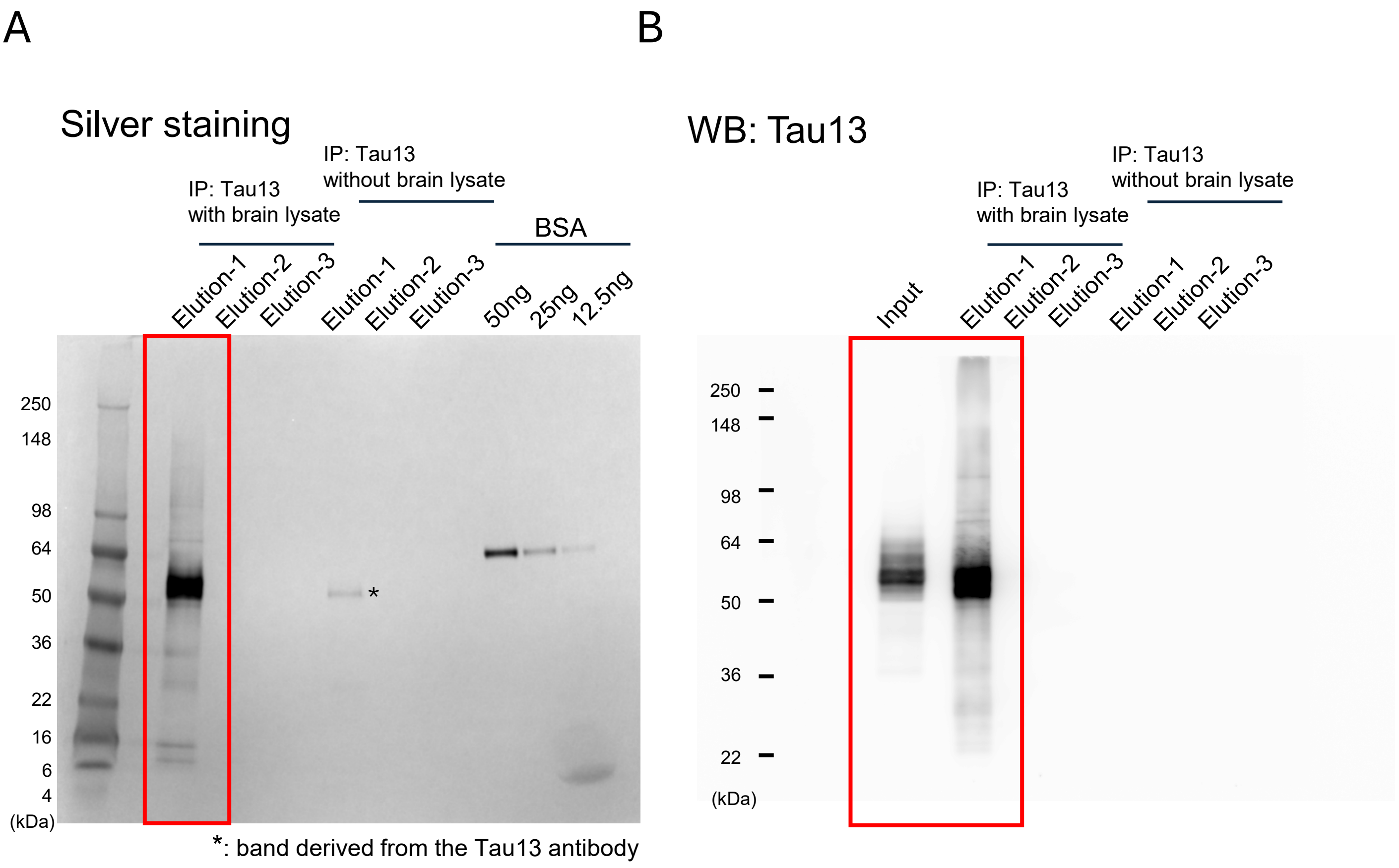

Supplementary Figure S1 Full view of the gel corresponding to Fig. 1A.
(A) Silver-stained gel showing the immunoprecipitation fractions with or without lysate. The red box indicates the region shown in Fig. 1A. Tau13 based immunoprecipitation was performed both in the absence and presence of brain lysate, and three sequential elution fractions were collected in each case. The three lanes on the right contain BSA standards of known concentrations. A band observed in elution 1 under the no-lysate condition (asterisk) is likely derived from the antibody itself. In contrast, under the lysate condition, a prominent band corresponding to tau was clearly observed. (B) Western blot using Tau13 antibody. The left lane shows the input, and the subsequent three lanes each represent sequential elution fractions prepared with or without lysate. The red box indicates the region shown in Fig. 1B.

Figure S2

		Found in Sample:							
Peptide ID	Modifications in Master Proteins	MAPT KI #1	MAPT KI #2	MAPT KI #3	MAPT KI #4	doubld KI #1	doubld KI #2	doubld KI #3	doubld KI #4
1	2xMethyl [R23(100); K24(100)]	Peak Found	High	Peak Found	Peak Found	Peak Found	Peak Found	Peak Found	Peak Found
2	1xMethyl [K24(100)]	High	High	High	High	High	High	High	High
3	1xAcetyl [K24(100)]	Peak Found	Peak Found	Peak Found	Peak Found	High	Peak Found	Medium	Peak Found
4	2xMethyl [K24(100); K44(100)]	High	High	High	High	High	High	High	High
5	2xMethyl [K24(100); K44(100)]	High	Peak Found	High	n/a	High	High	High	Peak Found
6	2xMethyl [K24(100); K44(100)]; 2xPhospho [T30(98.2); T50(98.2)]	High	Peak Found	High	n/a	High	Peak Found	High	Peak Found
7	1xMethyl [K44(100)]	High	High	High	Peak Found	High	High	Peak Found	High
8	1xPhospho [S113(100)]	High	High	High	Peak Found	High	High	High	High
9	1xMethyl [R155(100)]	Medium	Medium	Medium	Peak Found	Peak Found	Peak Found	Peak Found	Medium
10	1xAcetyl [K163(100)]	Medium	Medium	High	High	High	Medium	High	High
11	1xMethyl [K163(100)]	Peak Found	Peak Found	Peak Found	n/a	High	High	Peak Found	High
12	1xAcetyl [K174(100)]	High	High	High	Peak Found	High	Medium	High	High
13	1xPhospho [T175(100)]	High	High	Peak Found	High	High	High	High	High
14	1xPhospho [T181(100)]	High	High	Medium	High	High	High	High	High
15	2xPhospho [T175(100); T181(100)]	Medium	Peak Found	Medium	Peak Found	High	Peak Found	Medium	Medium
16	1xPhospho [T181(100)]	High	High	High	High	High	High	High	High
17	1xPhospho [T181(100)]	High	High	High	High	High	High	High	High
18	2xPhospho [T181(100); S/T]	n/a	n/a	Peak Found	n/a	Medium	Peak Found	High	n/a
19	1xPhospho [T181(100)]	n/a	n/a	n/a	High	n/a	n/a	n/a	n/a
20	1xPhospho [T181(100)]	High	High	High	Peak Found	High	High	High	High
21	2xPhospho [S198(98.4); S199(98.5)]	Peak Found	Peak Found	Peak Found	Peak Found	High	Peak Found	Medium	Peak Found
22	1xPhospho [S202(100)]	High	High	High	Peak Found	High	High	High	High
23	1xPhospho [S202(100)]	High	High	High	High	High	High	High	High
24	2xPhospho [S202(100); T205(99.4)]	High	High	Peak Found	Medium	High	High	High	High
25	1xMethyl [R209(99.4)]	High	Peak Found	High	High	Peak Found	High	Peak Found	Peak Found
26	1xPhospho [S214(99.5)]	High	High	High	High	High	High	High	High
27	1xPhospho [S214(100)]	High	High	High	Medium	High	High	High	High
28	1xPhospho [S214(99.6)]	Medium	High	High	High	High	High	High	High
29	1xPhospho [T217(100)]	Medium	Peak Found	Peak Found	Peak Found	High	High	Medium	Medium
30	1xAcetyl [K225(100)]	High	Medium	High	Medium	High	High	High	Peak Found
31	1xPhospho [T231(100)]	High	High	High	Medium	High	Medium	High	High
32	1xPhospho [T231(100)]	Peak Found	Peak Found	Medium	Peak Found	High	Medium	Medium	Peak Found
33	1xPhospho [T231(100)]	High	High	High	High	High	High	High	High
34	2xPhospho [T231(100); S235(99.5)]	High	High	High	Medium	High	Medium	High	High
35	1xMethyl [K259(100)]	High	High	High	High	High	High	High	High
36	1xPhospho [S262(99.6)]	Medium	High	Peak Found	Peak Found	High	High	High	Medium
37	1xAcetyl [K259(100)]	Peak Found	Peak Found	Peak Found	Peak Found	High	Peak Found	Peak Found	Peak Found
38	1xMethyl [K259(100)]	High	High	High	High	High	High	High	High
39	1xPhospho [S262(100)]	High	High	High	High	High	High	High	High
40	1xPhospho [S262(100)]	High	High	Medium	Peak Found	Medium	Peak Found	Medium	High
41	1xPhospho [S262(99.6)]	High	High	High	High	High	High	High	High
42	1xAcetyl [K290(100)]	Peak Found	Peak Found	High	Peak Found	High	Peak Found	Peak Found	High
43	1xAcetyl [K311(100)]	High	High	Peak Found	Peak Found	High	High	Peak Found	High
44	2xMethyl [K311(100); K317(100)]	High	High	High	High	High	Medium	High	High
45	1xAcetyl [K321(100)]	Peak Found	High	Peak Found	Peak Found	High	High	High	High
46	1xPhospho [S324(100)]	Peak Found	Peak Found	Peak Found	High	High	Peak Found	Peak Found	High
47	1xAcetyl [K331(100)]	n/a	Peak Found	Peak Found	n/a	Peak Found	Peak Found	High	Peak Found
48	1xAcetyl [K343(100)]	Peak Found	Peak Found	Peak Found	High	Peak Found	Peak Found	Peak Found	Peak Found
49	1xMethyl [R349(100)]	Medium	Medium	Medium	Peak Found	High	Medium	High	Medium
50	1xAcetyl [K353(100)]	Peak Found	Peak Found	High	Peak Found	Peak Found	Peak Found	High	High
51	1xPhospho [S356(100)]	High	High	High	High	High	High	High	High
52	1xAcetyl [K369(99.6)]	High	High	High	Peak Found	High	High	High	High
53	1xPhospho [S356(100)]	High	High	High	High	High	High	High	High
54	1xMethyl [K385(100)]	Peak Found	High	High	Peak Found	Peak Found	High	High	High
55	1xAcetyl [K385(100)]	Peak Found	Peak Found	Peak Found	Peak Found	High	Peak Found	Peak Found	Peak Found
56	1xPhospho [S396(100)]	High	High	High	High	High	High	High	High
57	2xPhospho [S396(100); T403(99.3)]	High	High	High	High	High	High	High	High
58	3xPhospho [S396(100); S400(100); S404(100)]	High	High	High	High	High	High	High	High
59	2xMethyl [K385(100); K395(100)]	Peak Found	Peak Found	Medium	Peak Found	Peak Found	Peak Found	Medium	Peak Found
60	2xMethyl [K385(100); K395(100)]; 2xPhospho [S396(98.7); T/S/Y]	Peak Found	n/a	n/a	Medium	Medium	Peak Found	n/a	n/a
61	1xPhospho [S400(100)]	High	High	High	High	High	High	High	High
62	2xPhospho [S396(100); S400(100)]	High	High	High	High	High	High	High	High
63	3xPhospho [S396(100); S400(100); S404(100)]	High	High	Medium	High	High	High	High	High
64	1xPhospho [S400(100)]	High	High	High	High	High	High	High	High
65	6xPhospho [S413; T/S]	High	Peak Found	Medium	Peak Found	n/a	n/a	n/a	High
66	1xAcetyl [K438(100)]	Peak Found	Peak Found	Medium	Peak Found	Peak Found	Peak Found	Medium	High
67	1xPhospho [S416(98.8)]	Peak Found	n/a	Peak Found	High	Peak Found	Peak Found	Peak Found	Peak Found

Supplementary Figure S2 Summary of all tau peptides and post-translational modifications (PTMs) identified by LC–MS/MS.

This table lists all peptides detected across individual samples, including those containing post-translational modifications. For each peptide, the modification type and modified residue(s) identified in the "Modifications in Master Proteins" field (e.g., 2 × Methyl [R23(100); K24(100)]) indicate the PTMs assigned by the search engine, with site localization probabilities shown in parentheses. Detection levels in each individual mouse are categorized as High, Medium, Peak found, or n/a (not detected), based on the confidence score and extracted ion chromatogram (XIC) intensity. Because identical PTMs were occasionally detected on multiple overlapping peptides, unique peptide IDs were assigned for clarity and to avoid ambiguity in cross-sample comparison.This table provides a comprehensive overview of all PTM-containing and unmodified peptides used for comparative analyses in this study.

Figure S3

A

```
tctgcctttc ctcttctctc tcctcctctc tcatctccag gtgcaaatag
tctacaaacc agttgacctg agcaaggtga cctccaagtg tggctcatta
ggcaacatcc atcatcaacc aggtagccct gtggaaggtg agggttggga
cgggagggtg caggggggtgg aggagtcctg gtgaggctgg aac
```

B

injection	litter size	indel	with the K331Q mutation
250	76	57	5

Supplementary Figure S3 Generation of *MAPT*^{K331Q} knockin mice

(A) Sequence of the ssODN used for generating the K331Q KI mice. The region underlined in blue indicates the sgRNA target sequence. The red base "c" corresponds to the edited nucleotide, which is "a" in the wild-type sequence. This AAA→CAA substitution results in a lysine-to-glutamine (Lys→Gln) replacement at position 331.(B) Summary of the genome-editing outcomes, including the number of fertilized eggs injected, the number of pups born, the number of indels obtained, and the number of mice carrying the K331Q mutation.

Figure S4

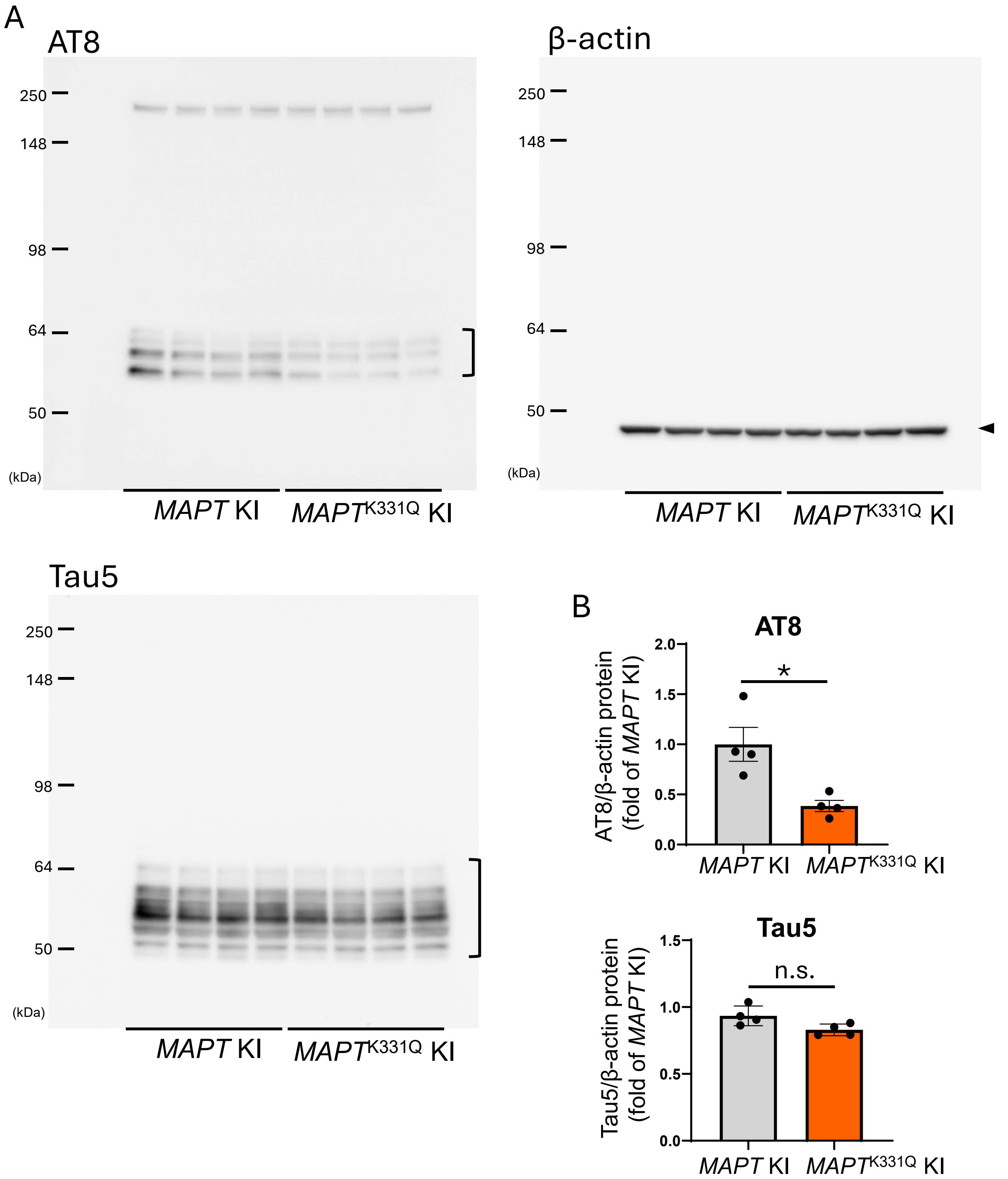

Supplementary Figure S4 Phosphorylation state of tau in soluble fractions of 18-month-old *MAPT* KI and *MAPT*^{K331Q} KI mice.

(A) Western blotting of Tris-HCl soluble fractions prepared from cortices of 18-month-old *MAPT* KI and *MAPT*^{K331Q} KI mice (n = 4 per genotype; all males). Blots were probed (from top to bottom) with Tau5 (total tau), AT8 (phosphorylated tau at Ser202/Thr205), and β-actin (loading control). (B) Quantification of western blot signals shown in (A). Data are presented as mean ± SEM. *$p < 0.05$. n.s.: not significant

Figure S5

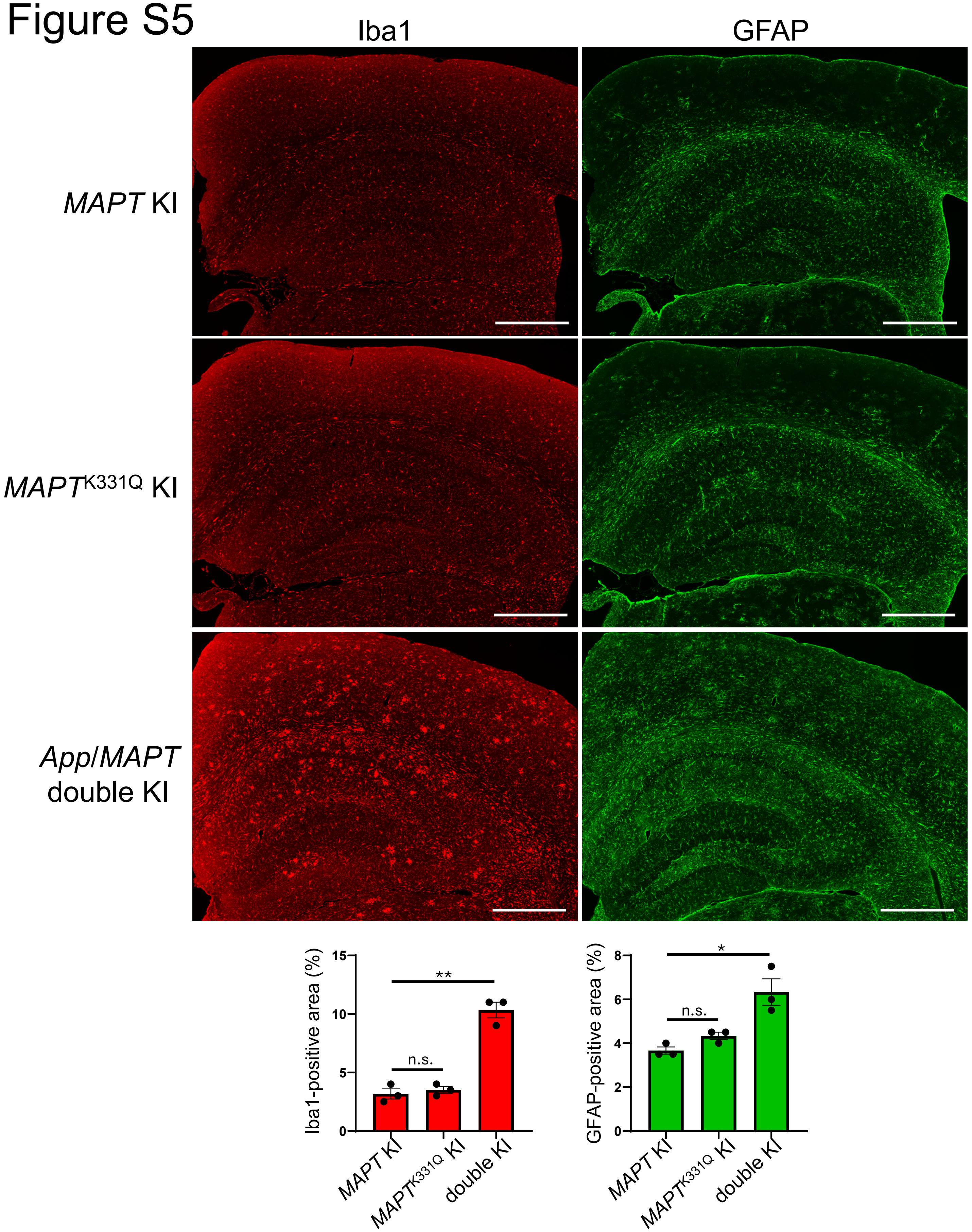

Supplementary Figure S5 Immunohistochemical analysis of microglial and astrocytic reactivity at 24 months of age. Representative immunofluorescence images of cortical brain sections from 24-month-old *MAPT* KI, *MAPT*^{K331Q} KI, and *App*/*MAPT* double KI mice stained for Iba1 (red) and GFAP (green). In *App*/*MAPT* double KI mice, prominent microglial and astrocytic reactivity was observed in association with amyloid pathology. In contrast, no apparent differences in Iba1 or GFAP immunoreactivity were detected between *MAPT* KI and *MAPT*^{K331Q} KI mice. Scale bar, 500 µm. Quantification is shown below (n = 3 mice per genotype; two sections quantified per mouse), representing the percentage of Iba1- or GFAP-positive area within a fixed cortical ROI. n.s., not significant; *p < 0.05; **p < 0.01.

Figure S6

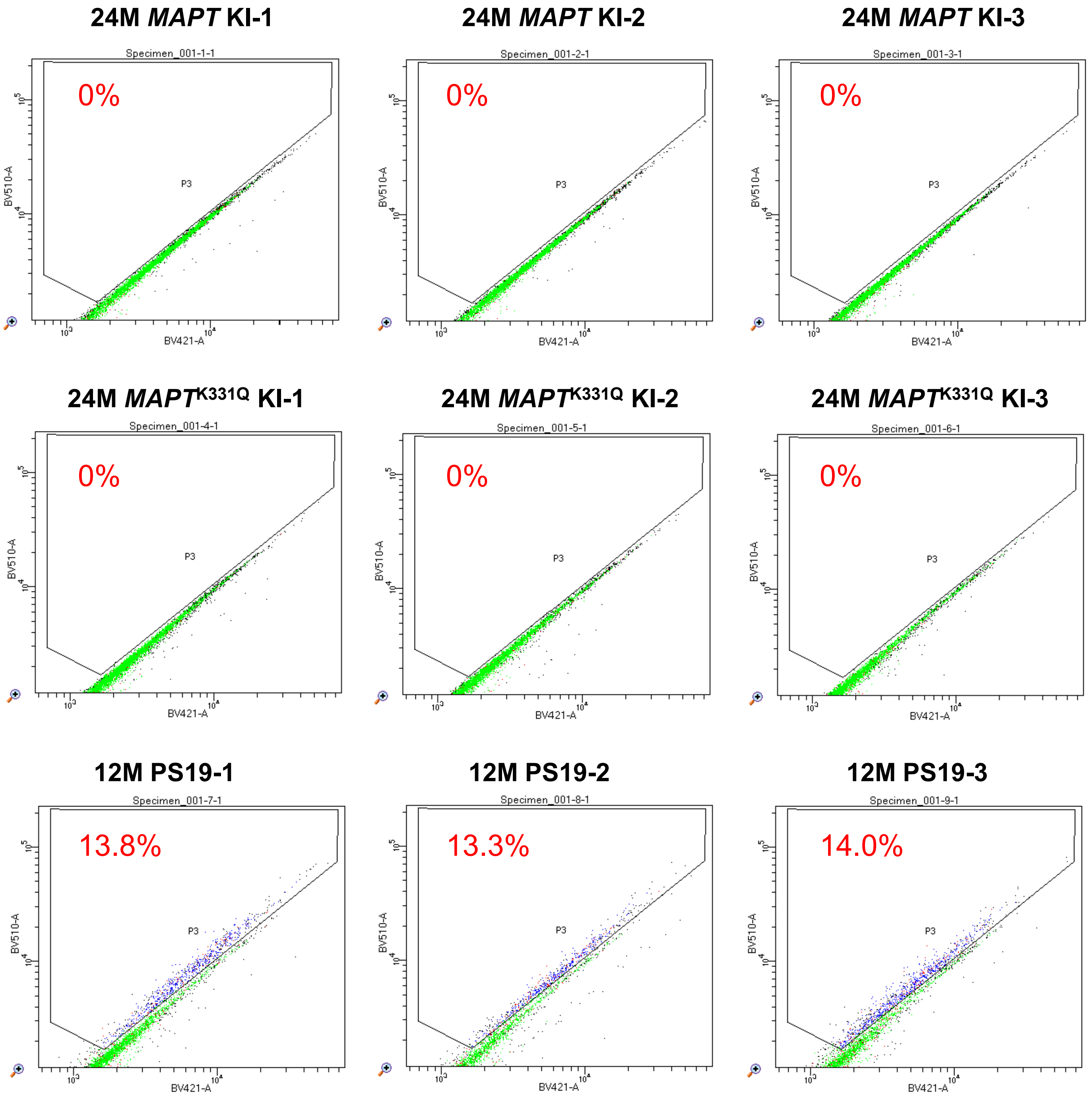

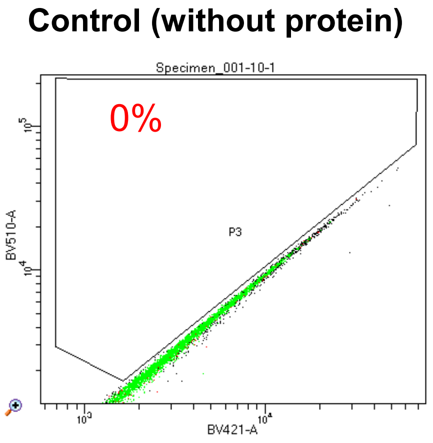

Supplementary Figure S6 Flow cytometry analysis of tau seeding activity.
FACS plots of biosensor cells transfected with brain-derived proteins from all individual samples. The vertical axis indicates FRET-positive cells and the horizontal axis indicates CFP-positive cells. The percentage of cells within the gated region is shown for each sample. The bar graph in Fig. 5C summarizes these percentages.

Figure S7

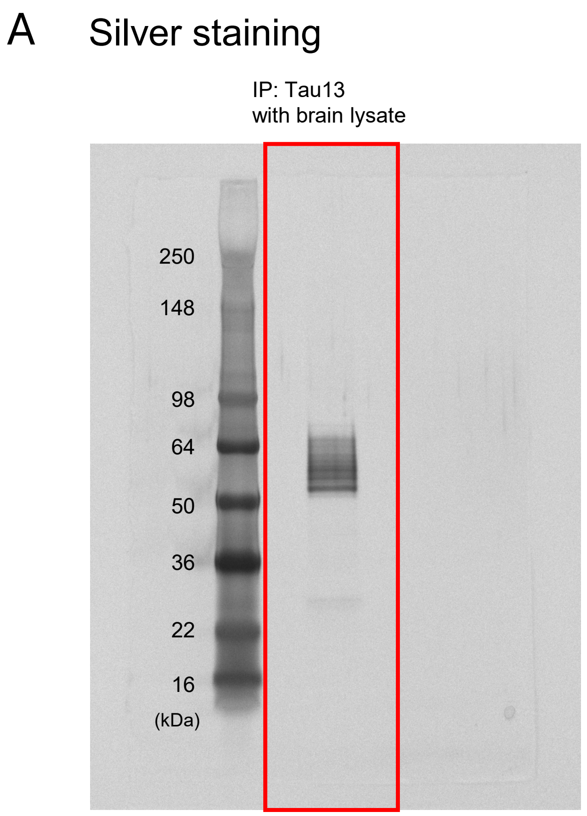

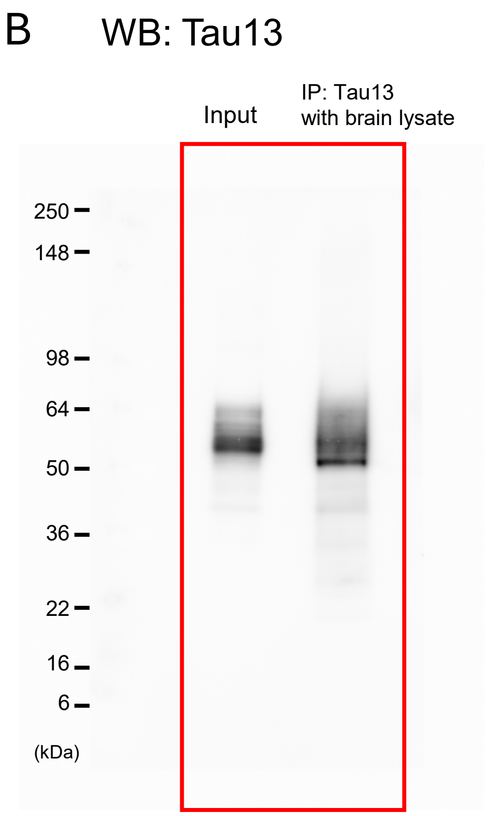

Supplementary Figure S7 Full Western blot images corresponding to Figure 3A and B
(A, B) This panel shows the full, uncropped image of the silver staining and the Western blot presented in Figure 3A and B. The area outlined in red corresponds to the region shown in Figure 3.

Figure S8

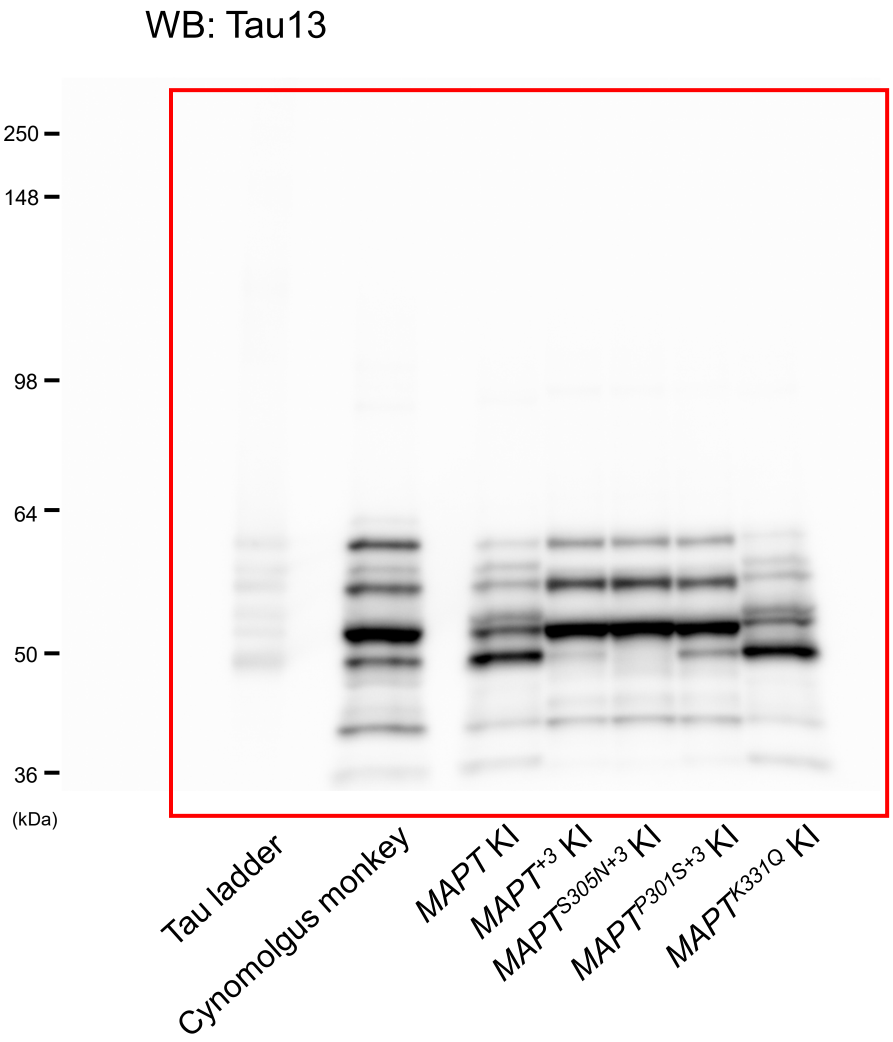

Supplementary Figure S8 Full Western blot images corresponding to Figure 4C

This panel shows the full, uncropped image of the Western blot presented in Figure 4C. The area outlined in red corresponds to the region shown in Figure 4C.

Figure S9

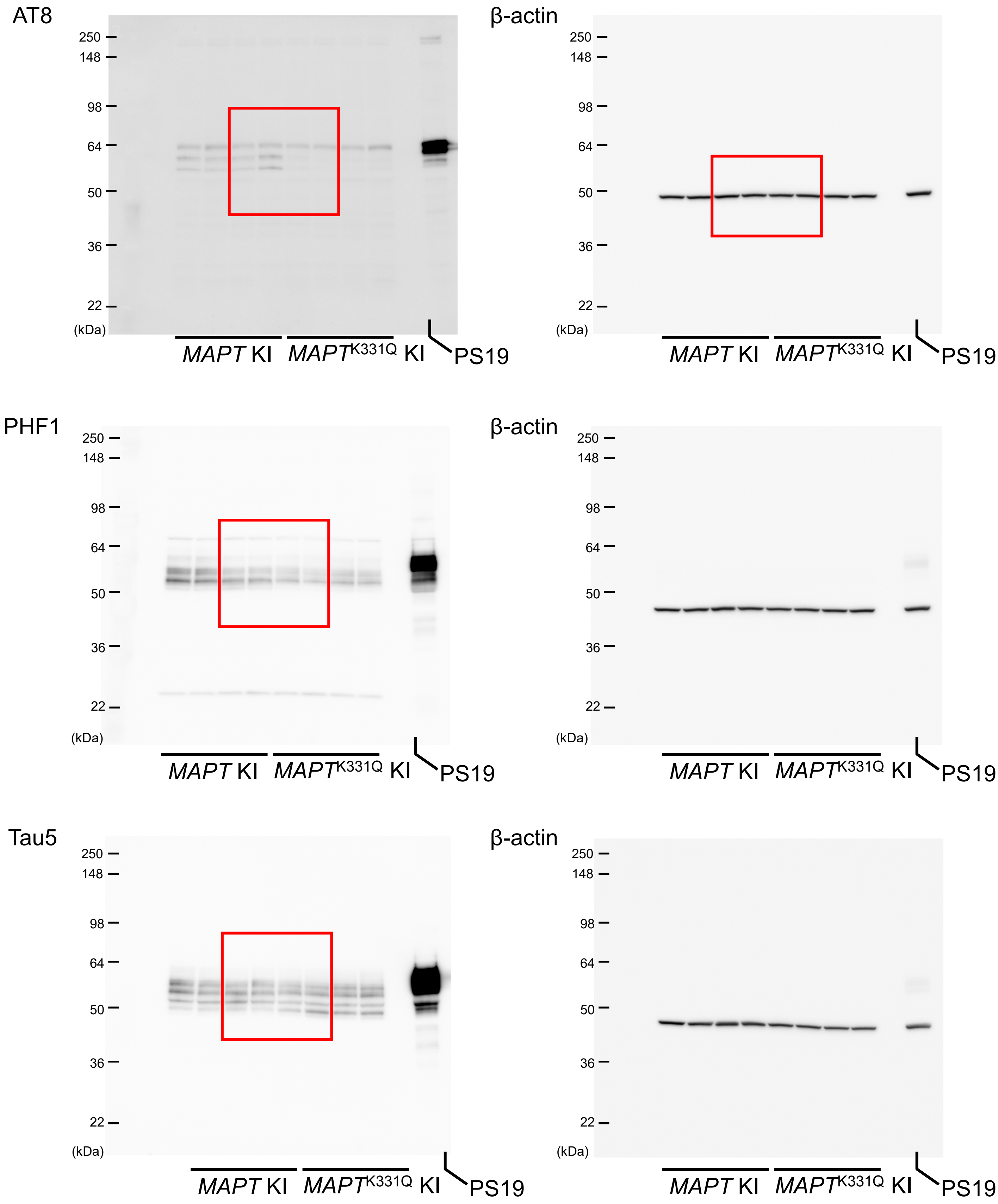

Supplementary Figure S9 Full Western blot images corresponding to Figure 5A

This panel shows the full, uncropped image of the Western blot presented in Figure 5A. The area outlined in red corresponds to the region shown in Figure 5A. he corresponding β-actin blots for each membrane are shown on the right.

Figure S10

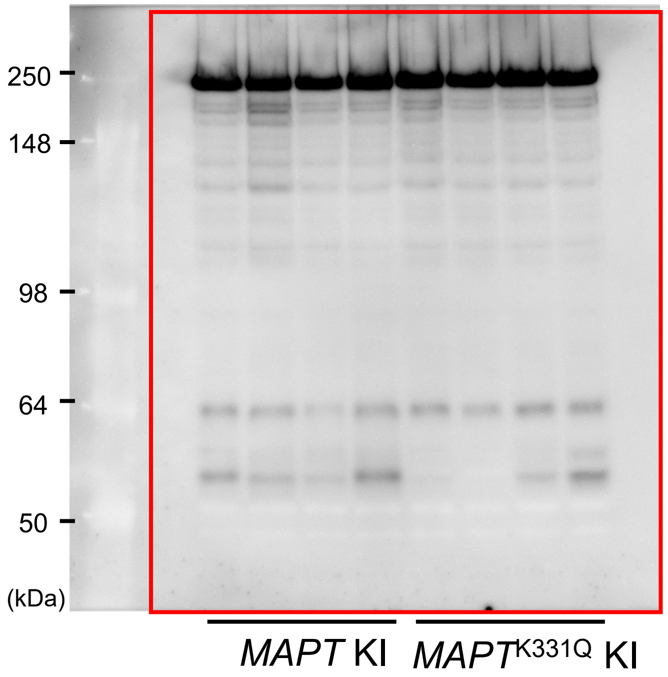

Supplementary Figure S10 Full Western blot images corresponding to Figure 5C
This panel shows the full, uncropped image of the Western blot presented in Figure 5C. The area outlined in red corresponds to the region shown in Figure 5C.
